# Efficacy and Haematologic Toxicity of Palliative Radioligand Therapy of Metastatic Castrate-Resistant Prostate Cancer with Lutetium-177-Labeled Prostate-Specific Membrane Antigen in Heavily Pre-Treated Patients

**DOI:** 10.3390/diagnostics11030515

**Published:** 2021-03-14

**Authors:** Murali Kesavan, Danielle Meyrick, Marat Gallyamov, J. Harvey Turner, Sharon Yeo, Giuseppe Cardaci, Nat P. Lenzo

**Affiliations:** 1 Department of Haematology, School of Medicine, The University of Western Australia, Perth 6009, Australia; murali.kesavan@research.uwa.edu.au; 2 Department of Nuclear Medicine, School of Medicine, The University of Western Australia, Perth 6009, Australia; danielle.meyrick@genesiscare.com (D.M.); John.Turner@health.wa.gov.au (J.H.T.); 3 GenesisCare, East Fremantle, Fremantle 6158, Australia; marat.gallyamov@genesiscare.com (M.G.); Sharon.Yeo@genesiscare.com (S.Y.); 4 School of Medicine, The University of Notre Dame, Fremantle 6160, Australia; jcardaci@dni.com.au

**Keywords:** LuPSMA, mCRPC, hematologic toxicity, MDS

## Abstract

Background: Metastatic castration-resistant prostate cancer (mCRPC) remains a significant contributor to the global cancer burden. lutetium-177-prostate-specific membrane antigen radioligand therapy (^177^Lu-PSMA RLT) is an effective salvage treatment. However, studies have highlighted haematologic toxicity as an adverse event of concern. We report our single-centre experience of compassionate access palliative ^177^Lu-DOTAGA-(I-y)fk(Sub-KuE) (^177^Lu-PSMA I&T) with respect to efficacy and haematologic safety. Methods: Patients with mCRPC and adequate bone marrow/liver function were included. All patients included underwent baseline and response assessment by Gallium-68-PSMA-11 positron emission tomography/computed tomography (^68^Ga-PSMA-11 PET/CT). Prescribed activity of therapy was a median 6.24 GBq per patient per cycle (IQR1.29 GBq), administered in 8-week intervals, up to four cycles. Response was assessed by prostate specific antigen (PSA) and a week-12 PET/CT. Incidence of grade ≥ 3 haematologic toxicity, including association with risk factors (age ≥ 70 years, prior/concurrent therapy, presence of metastases, and number of cycles completed), was analysed. Results: One hundred patients completed one cycle of ^177^Lu PSMA I&T and underwent response assessment by both PSA and PET/CT. Two patients had an uninterpretable week-12 PET/CT. Median age was 70 (50–89), median number of prior therapies was three (1–6), and median follow up was 12-months. Fifty-four percent achieved a PSA response. Disease control rate (DCR) by PET/CT was 64% (29% SD, 34% PR, and 1% CR). Disease control by PET/CT was associated with an improved one-year overall survival (OS) compared to non-responders, median OS not-reached vs 10-months (*p* < 0.0001; 95% CI: 0.08–0.44). Regarding haematologic toxicity, 11% experienced a grade ≥ 3 cytopenia (self-limiting). No cases of myelodysplasia/acute leukaemia (MDS/AL) have been recorded. No association with risk factors was demonstrated. Conclusion: ^177^Lu-PSMA I&T is a safe and effective palliative outpatient treatment for mCRPC. ^68^Ga-PSMA-11 PET/CT response is associated with an improved one-year OS and may be used to adapt therapy.

## 1. Introduction

Prostate cancer is the second most common cancer diagnosis in men and the fifth leading cause of death worldwide [1]. Androgen deprivation therapy (ADT), either surgical or chemical, remains the mainstay of initial therapy. Following on from the success of first-generation anti-androgen therapies, numerous studies have demonstrated the efficacy of second-generation agents, including abiraterone [2,3], enzalutamide [4,5], and apalutamide [6], as single agents and in combination with other modalities [7] for metastatic castration-resistant prostate cancer (mCRPC). Despite the demonstrated efficacy of antiandrogen-based salvage, the vast majority of patients will become resistant to ADT, developing advanced mCRPC. Although feasible, the limited overall efficacy of salvage chemotherapy and the associated burden on patients and health systems limits its utility. Thus, there remains an urgent unmet need for a safe, tolerable, and cost-effective non-chemotherapy-based approach for advanced mCRPC.

Identification of prostate-specific membrane antigen (PSMA) has enabled the development of targeted immunotherapeutic strategies for CRPC. A type II membrane protein PSMA is present on prostate epithelial cells and proportionally overexpressed in advanced prostate carcinoma, making it an ideal target for radioligand therapy (RLT) [8]. PSMA targeting has rapidly evolved with the development of accurate tumour-targeted imaging with Gallium-68-PSMA imaging and therapy based on the findings on positron emission tomography/computed tomography (^68^Ga-PSMA-11 PET/CT) and RLT using a number of alternative PSMA ligands labelled with lutetium-177 (^177^Lu-PSMA RLT). These advances have yielded improved patient selection and tumour targeting [8].

Results from the phase III TheraP study comparing ^177^Lu-PSMA RLT to cabazitaxel chemotherapy have reported a superior response rate (66% for RLT versus 37% for chemotherapy), however, ^177^Lu-PSMA RLT was associated with a higher incidence of grade ≥ 3 haematologic toxicity and overall grade 5 adverse events [9], substantiating previously reported concern for haematologic toxicity in heavily pre-treated patients [10,11]. As indications for PSMA-RLT expand, there must be a continued effort to optimize the haematologic safety and efficacy of treatment.

Since 2015, our centre has offered compassionate access to palliative ^177^Lu-DOTAGA-(I-y)fk(Sub-KuE) (^177^Lu-PSMA I&T) therapy for patients with progressive mCRPC. In this short communication, we focus on analysis of the efficacy and haematologic safety of ^177^Lu-PSMA I&T in 100 consecutively treated mCRPC patients.

## 2. Materials and Methods

### 2.1. Patients

Consecutive patients with known progressive mCRPC (as per Prostate-Specific Antigen Working Group [PCWG3] [12]) criteria were prospectively reviewed and treated between November 2015 and August 2018. All patients underwent baseline ^68^Ga-PSMA-11 PET/CT to confirm PSMA avid disease. Detailed inclusion and exclusion criteria have been previously presented [13]. In brief, patients treated were required to have a European Cooperative Oncology Group (ECOG) performance status ≤ 2, adequate bone marrow function (haemoglobin ≥ 90 g/L, platelet ≥ 100 × 10^9^/L and neutrophils ≥ 1 × 10^9^/L), and serum bilirubin ≤ 1.5 times the upper limit of normal (ULN). Patients with a history of prior radiotherapy to >25% of skeleton, prior strontium-89 or samarium-153 therapy, other active cancers, or clinically significant organ dysfunction were excluded. Given the renal safety of ^177^Lu-PSMA RLT, patients with renal impairment were not specifically excluded. Concomitant hormone therapy was permitted in selected cases at the discretion of the treating oncologist. The local institutional review board (IRB) and Human Research Ethics Committee (HREC) approved this research (HREC: HPH474). Written informed consent was obtained from all patients before therapy. Therapy was administered in compliance with the Australian Therapeutic Goods Administration Special Access Scheme regulations for compassionate usage.

### 2.2. Treatment and Response Assessment

Lutetium-177-PSMA I&T was produced by a qualified radiochemist in an in-hospital laboratory, operating under good practice conditions and in compliance with published guidelines [14]. Quality control of the ^177^Lu-PSMA I&T product was performed by thin-layer and high-performance liquid chromatography [13]. The prescribed activity of therapy was a median of 6.1 GBq (IQR 1.3 ) per patient per cycle and was administered at 8-week intervals. Subsequent cycles were only prescribed upon demonstration of adequate bone marrow recovery (haemoglobin ≥ 90 g/L, platelet ≥ 100 × 10^9^/L, and neutrophils ≥ 1 × 10^9^/L) and clinical recovery as determined by the patients’ treating oncologist. Biochemical response was confirmed by repeat prostate-specific androgen (PSA) prior to each cycle and ^68^Ga-PSMA-11 PET/CT response was assessed at week 12 post day one of cycle one. PSA response was defined according to current Prostate Cancer Working Group (PCWG3) criteria with response defined as a value ≥50% below baseline and progression defined as an increase that is ≥25% and ≥2 ng/mL above the nadir, both confirmed by a second value ≥3 weeks later. Imaging response was defined according to PERCIST, EAU, and EANM consensus criteria: complete response (CR) as the disappearance of any lesion with tracer uptake; partial response (PR) as reduction of uptake and tumour PET volume by >30%; stable disease (SD) as change of uptake and tumour PET volume ± ≤30% without evidence of new lesions; progressive disease (PD) as appearance of >2 new lesions or increase of uptake or tumour PET volume ≥30% [15,16]. The disease control rate (DCR) was defined as the percentage of patients achieving CR, PR, or SD based on PET/CT response assessment.

### 2.3. Haematologic Toxicity and Survival Analysis

Toxicities were graded according to the National Cancer Institute Common Terminology Criteria for Adverse Events (NCI CTCAE v4.03). Specific haematologic risk factors analysed were based on previously published reports [10,14] and included age ≥70 years, prior radiotherapy, prior chemotherapy, presence of bone (>15) and nodal metastases, cumulative activity of therapy, and number of cycles of ^177^Lu-PSMA I&T therapy completed. Non-parametric correlation using Chi-square testing was performed to assess significance of risk factors with respect to advanced grade haematologic toxicity using both univariable and multivariable analyses. Binary logistic regression analysis was used to assess the risk of total cumulative activity. Survival comparisons were made using Kaplan–Meier methodology with progression-free and overall survival (PFS and OS) calculated from the date of day one of cycle one of ^177^Lu-PSMA I&T. Survival curves were compared using the Log-rank test. All statistical analysis was performed using IBM SPSS statistics for Windows version 26 (IBM Corp., Armonk, N.Y., USA).

## 3. Results

A total of 100 mCRPC patients completed ≥1 cycles of ^177^Lu-PSMA I&T therapy and had response assessment by PSA and PET/CT. Two patients had uninterpretable follow up PET/CT results. At the time of analysis, the median duration of follow up was 12-months (range 3–38). The median age of patients was 70-years (range 50–89), with a median PSA of 73 ng/mL (range 0.1–5000). All patients had confirmed metastatic disease (61% combined nodal and bone) and were relapsed/refractory following prior surgical and or chemical castration therapy with a median number of prior lines of therapy of three (range 1–6), including prior abiraterone (40%), enzalutamide (48%), and bicalutamide (36%). Other prior therapies included chemotherapy (57%), palliative radiotherapy (65%), radium (4%), and peptide-receptor-radionuclide-therapy (PRRT) (2%). Detailed patient characteristics are presented in Table 1.

### 3.1. Response Assessment

**Figure 1:**
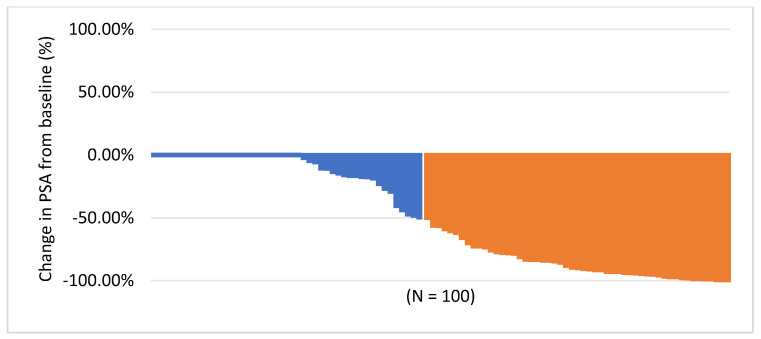
Best PSA response. Prostate-specific-antigen (PSA) response defined as PSA value ≥ 50% below baseline [12].

Of the 100 patients analysed, the best PSA response of ≥ 50% reduction from baseline was documented in 53 (53%) patients (Figure 1).

**Figure 2:**
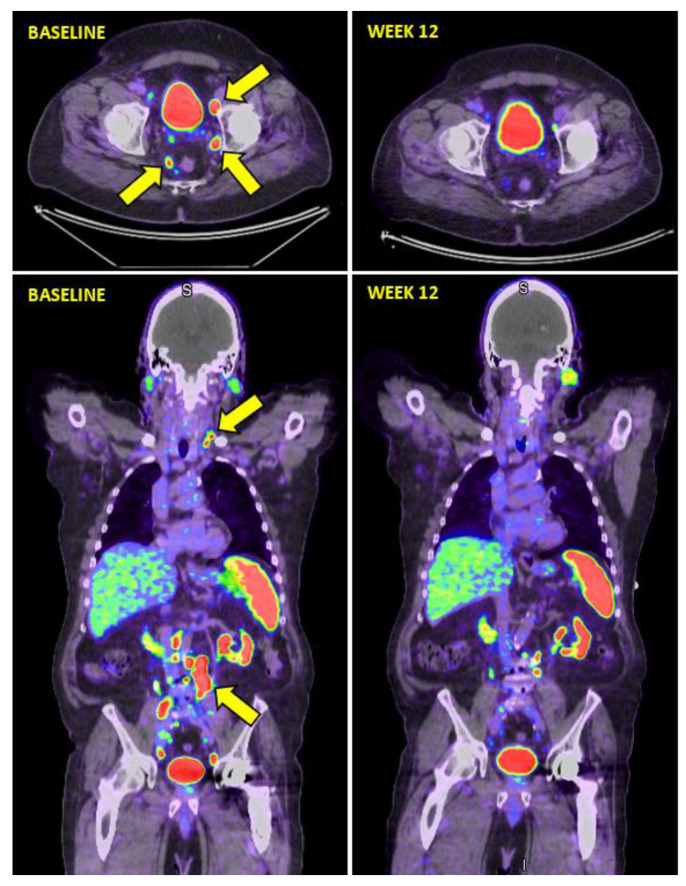
Example of week-12 ^68^Ga-PSMA-11 PET/CT response assessment following ^177^Lu-PSMA I&T for mCRPC. Baseline ^68^Ga-PSMA-PET/CT (left panels) nodal metastases involving left cervical chain and multiple abdominopelvic nodes (yellow arrows). Week-12 ^68^Ga-PSMA-PET/CT response assessment (right panels) showing a near complete response with resolution of the majority of nodal disease above and below the diaphragm. Consistent with an excellent partial remission.

The DCR by week-12 ^68^Ga-PSMA-11 PET/CT was 64% (29% SD; 34% PR; and 1% CR) (Figure 2).

At the time of analysis, 86 (86%) patients had documented PSA progression, of whom 52 (60%) had concurrent evidence of PD on their week-12 ^68^Ga-PSMA-11 PET/CT (three patients had progression by imaging but not PSA). Regarding survival outcomes, the median overall PFS (by PSA progression) was 6-months (95% CI; 4.11–7.89), and the median overall OS has not been reached.

**Figure 3:**
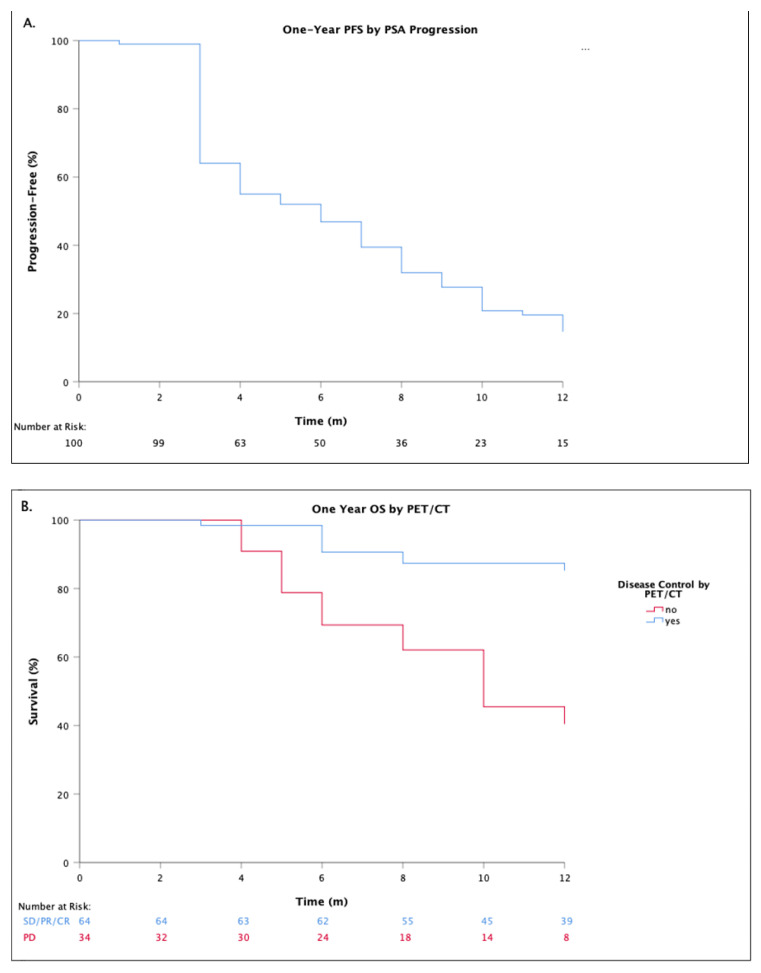
Survival outcomes. (**A**) One-year progression-free-survival (PFS), by prostate specific antigen (PSA) measurement, with progression defined as a >25% increase in PSA and >2 ng/mL above nadir, confirmed by progression at two timepoints at least 3 weeks apart; (**B**) One-year overall-survival (OS) stratified by week-12 Gallium-68 prostate specific membrane antigen positron emission tomography/computed tomography (PET/CT) based response. Disease control defined as any patient achieving stable disease (SD), partial-response (PR), or complete response (CR) as per PERCIST, EAU, and EANM consensus criteria [14,15].

Twenty-nine (29%) deaths have been recorded, all due to disease related events. This includes all patients with documented progression by combined PSA measurement and ^68^Ga-PSMA-11 PET/CT. Disease control demonstrated by week-12 ^68^Ga-PSMA-11 PET/CT was associated with a significantly improved one-year OS when compared to patients with progression; median OS not yet reached versus 10-months respectively (HR 0.19; *p* < 0.0001; 95% CI: 0.08–0.44) (Figure 3).

This survival benefit was sustained irrespective of the total number of cycles of therapy completed by individual patients.

### 3.2. Haematologic Toxicity

All patients had adequate data available for assessment of haematologic toxicity. Eleven (11%) patients experienced a grade ≥3 haematologic toxicity; two patients presented with anaemia, five with lymphopenia, one with neutropenia, and three with combined anaemia and thrombocytopenia. Toxicity was self-limiting without necessity for admission. No statistically significant correlation between the defined risk factors and the development grade ≥3 haematologic toxicity was identified (Table 2).

In particular, prior exposure to both docetaxel and cabazitaxel was not associated with an increased risk of haematologic toxicity (odds ratio [OR], 0.63; 95% CI: 0.15 to 2.62; *p* = 0.69). Regression analysis failed to identify a statistically significant relationship between the cumulative activity of therapy and development of grade ≥3 haematologic toxicity (R^2^ 0.038; 95% CI: 0.80-1.05; *p* = 0.20). No cases of myelodysplasia/acute leukaemia (MDS/AL) have been observed.

## 4. Discussion

Our single-centre experience of compassionate access palliative ^177^Lu-PSMA I&T for mCRPC demonstrates the feasibility, safety, efficacy, and adaptability of outpatient RLT in heavily pre-treated patients. Despite its limitations, our study highlights a number of important points regarding the real-world applications of RLT for mCRPC.

The 11% incidence of advanced grade haematologic toxicity is similar to that reported in other studies [17,18,19], including the landmark single-arm Phase II Australian study of ^177^Lu-PSMA (mean 7.5 GBq 6-weekly) for mCRPC from Hofman et al., who observed a 13% incidence of grade ≥ 3 in their initial cohort [11]. The expanded follow up cohort study (*n* = 50) included heavily pre-treated patients, and similar to our results, reported a best PSA response in 64% (32/50) and objective response (CR and PR) of 56% (15/27). Most recently, preliminary results of the TheraP phase III study (^177^Lu-PSMA (*n* = 99) versus cabazitaxel (*n* = 101)) reported a 23% incidence of grade ≥3 haematologic toxicity at a similar median follow up. Patients in this study received a higher frequency and administered therapeutic activity (6-week intervals at 8.5 GBq per cycle) of ^177^Lu-PSMA, which may account for the increased incidence. Reassuringly, this study also reported a similar one-year median biochemical PFS (by PSA measurement) of approximately 6-months [9].

Our response results are also comparable with that reported in the German pre-VISION single-centre analysis, comparing 6.0 Gbq versus 7.5 Gbq of mean administered therapeutic activity (*n* = 78). This retrospective analysis reported the best PSA responses of 35% and 54% and a superior median biochemical PFS of 9.5 vs 12.3, respectively, for the 6.0 Gbq and 7.5 Gbq cohorts [20]. Of note, the incidence of grade ≥3 haematologic toxicity was significantly higher in this study, greater than 20% incidence in both cohorts (predominantly manifesting as anaemia). This difference may be accounted for by previous chemotherapy exposure (100% of cases) and the short median therapy interval of 6.5 weeks for the 7.5 GBq group [20]. Although not specifically reported in this study, transfusion support would widely be considered as a standard of care for patients experiencing grade ≥3 anaemia [21]. The true efficacy and safety of a mean 7.5 GBq administered activity in chemo-refractory mCRPC will be addressed in the forthcoming phase III VISION study [22].

The self-limiting nature of toxicity in our study, without any reported incidence of persistent haematologic toxicity or MDS/AL, supports the haematologic safety of our flexible therapy protocol. This is an important observation given the increasing use of ^177^Lu-PSMA to treat mCRPC patients who have failed multiple lines of alkylating chemotherapy. No significant association was demonstrated between baseline patient factors, therapy related factors, and incidence of grade ≥3 haematologic toxicity, supporting published patient selection criteria [14]. As noted, the lack of association between risk factors analysed and haematologic toxicity in our study may relate to the comparatively lower administered activity of therapy (mean of 6.0 GBq administered in 8-week intervals), increased length of time per cycle allowing for more complete marrow recovery and relatively short duration of follow up with respect to secondary MDS/AL. It also highlights the limited predictive value of such factors for patients receiving RLT. In this regard, there are two advantages to the current protocol that must be considered for prospective patients in this setting. One is the monitoring of blood counts and delay of therapy until adequate bone marrow recovery, and the second is avoiding unnecessary over-treatment by limiting further exposure where an objective response has been attained.

As noted, there are a number of limitations to our study, primarily owing to the compassionate nature of therapy. First, the data presented is retrospective in nature and from a small heterogenous cohort of patients. Second, the limited duration of follow up precludes the drawing of any conclusions regarding the long-term efficacy of therapy. However, given the overall limited prognosis faced by mCRPC patients and palliative intent of therapy, the one-year survival data presented is relevant and clinically meaningful information for physicians and patients.

The ultimate goal of therapy for advanced CRPC is to improve a patient’s quality-of-life whilst providing disease control. The ideal therapy should therefore avoid individual clinical and/or financial toxicity [23]. Outpatient ^177^Lu-PSMA I&T lends itself to such an approach. Treatment is easily administered, and monitoring of routine blood counts and biochemistry enables efficient assessment of toxicity and response, enabling an adaptive approach, without apparent compromise in efficacy.

Finally, given the sensitivity and specificity of ^68^Ga-PSMA-11 PET/CT and limitations of PSA [24,25], the optimal method for patient monitoring in the setting of relapsed/refractory metastatic CRPC remains undefined. Within the context of PSMA RLT, the therapeutic utility of ^68^Ga-PSMA-11 PET/CT strongly supports its routine use in order to facilitate adaptive approaches for mCRPC patients.

## 5. Conclusions

Outpatient ^177^Lu PSMA I&T is a safe, effective, and adaptable treatment for advanced, heavily pre-treated mCRPC. Haematologic toxicity is modest and self-limiting. Interim ^68^Ga-PSMA-11 PET/CT response at 12 weeks post commencement of therapy is associated with an improved one-year OS and should be considered for all prospective patients. Further studies are needed to establish the optimal timing and schedule (including response assessment) of ^177^Lu-PSMA RLT for advanced CRPC.

## Figures and Tables

**Table 1:** Patient characteristics.

	*n* = 100 (%)
Age (Years): Median (range)	70 (range 50–89)
Age (Years) ≥ 70	54 (54)
PSA (ng/mL): Median (range)	73 (range 0.1–5000)
Median Gleason Score	9 (range 6–10)
Metastases	100 (100)
Nodal	12 (12)
Bone (>15 lesions)	26 (26)
Nodal + Bone	61 (61)
Other	1 (1)
Number of Lines of Prior Therapy	
1	1 (1)
2	16 (16)
3	34 (34)
4	28 (28)
5	18 (18)
6	3 (3)
Primary Therapy	
Radical prostatectomy	42 (42)
ADT	23 (23)
EBRT	26 (26)
Chemotherapy + ADT	9 (9)
Prior Hormone Therapies *	
Abiraterone	40 (40)
Bicalutamide	36 (36)
Enzalutamide	48 (48)
Apalutamide	1 (1)
Nilutamide	1 (1)
Prior Chemotherapy	
Docetaxel	29 (29)
Cabazitaxel	5 (5)
Prior Docetaxel and Cabazitaxel	20 (20)
Mitoxantrone	3 (3)
Other Prior Therapies	
Palliative radiotherapy	65 (65)
Radium	4 (4)
PRRT	2 (2)
Concurrent Hormone Therapy	14(14)
Abiraterone	6 (6)
Enzalutamide	6 (6)
Bicalutamide	1 (1)
Other	1 (1)
Number of Cycles Completed	
1	1(1)
2	56 (43)
3	27 (27)
4	15 (15)
5	1 (1)

* Individual patients received sequential therapy with one or more of these agents. PSA; prostate-specific antigen, ADT; androgen deprivation therapy, EBRT; external beam radiotherapy, PRRT; peptide-receptor-radionuclide-therapy.

**Table 2:** Univariable and multivariable analyses of selected risk factors and their odds-ratios for the development of grade ≥3 haematologic toxicity following therapy with ^177^Lu-PSMA I&T for mCRPC.

	OR	95% CI	*p* Value
Univariable Analyses			
Age ≥ 70 Years	1.47	0.42–5.26	0.55
Prior Palliative Radiotherapy (skeletal)	0.38	0.08–1.85	0.21
Prior Chemotherapy	1.18	0.32–4.46	0.78
Prior Docetaxel and Cabazitaxel	0.63	0.15–2.62	0.69
Presence of Bone and Nodal Metastases	1.34	0.38–4.76	0.64
^177^Lu-PSMA I&T Cycles Completed			
1	**		
2	0.70	0.19–2.56	0.59
3	0.61	0.16–2.27	0.46
4	**		
Multivariable Analyses			
Age ≥ 70 years	1.68	0.43–6.53	0.45
Prior Chemotherapy	1.18	0.28–4.96	0.82
Prior Docetaxel and Cabazitaxel	0.61	0.10–3.54	0.57
Presence of Bone and Nodal Metastases	1.35	0.36–5.14	0.65

** Insufficient events for calculation of an odds-ratio.

## Data Availability

The datasets generated during and/or analysed during the current study are available from the corresponding author on reasonable request.

## Abbreviations

^68^Ga-PSMA-11 PET/CT	Gallium-68-PSMA-11 positron emission tomography/computed tomography
^177^Lu	Lutetium-177
^177^Lu-PSMA I&T	^177^Lu-DOTAGA-(I-y)fk(Sub-KuE)
ADT	Androgen deprivation therapy
AL	Acute leukaemia
EANM	European Association of Nuclear Medicine
EAU	European Association of Urology
EBRT	External beam radiotherapy
ECOG	European Cooperative Oncology Group
IRB	institutional review board
NCI-CTCAE	National Cancer Institute Common Terminology Criteria for Adverse Events
mCRPC	Metastatic castrate resistant prostate cancer
MDS	Myelodysplastic syndrome
OS	Overall survival
OR	odds ratio
PERCIST	PET response criteria in solid tumors
PET/CT	Gallium-68-PSMA-11 positron emission tomography/computed tomography
PCWG	Prostate Cancer Working Group
PFS	Progression-free survival
PSA	Prostate-specific antigen
PSMA	Prostate-specific membrane antigen
RLT	Radioligand therapy
